# Exploratory evaluation of local hyperthermia (44 ± 2 °C) in granuloma annulare: A pilot randomized controlled study

**DOI:** 10.1016/j.jdin.2026.02.007

**Published:** 2026-03-03

**Authors:** Pin-yu Wen, Cong-cong He, Li-shanjie Chen, Rui-qun Qi, Xing-Hua Gao

**Affiliations:** a Department of Dermatology, The First Hospital of China Medical University, Shenyang, China; b NHC and Ministry of Education Key Laboratory of Immunodermatology, National Joint Engineering Research Center for Diagnosis and Treatment of Immunologic Skin Diseases, The First Hospital of China Medical University, Shenyang, China; c Zhoushan Maternal and Child Care Hospital, Zhoushan, China

**Keywords:** efficacy, granuloma annulare, localized hyperthermia, randomized controlled trial, refractory, safety, treatment

**Capsule Summary**

• This study integrates local hyperthermia, a therapy used for other skin conditions, into the management of granuloma annulare.
• It suggests local hyperthermia could change practice as a potential new treatment for granuloma annulare, especially in cases refractory to existing therapies.

Granuloma annulare (GA) is a benign, noninfectious granulomatous dermatosis, typically presenting as annular erythematous papules or plaques on the dorsal aspects of the extremities or the trunk.^1^ Current therapeutic options are often suboptimal and associated with significant adverse effects, necessitating the development of novel strategies to enhance efficacy and minimize treatment-related morbidity. Local hyperthermia is a physical therapeutic modality that induces immunomodulatory effects by elevating tissue temperature.^2^^,^^3^ A case report of our group describing successful management of subcutaneous GA, highlighting its therapeutic potential.^4^

To investigate the potential of localized hyperthermia at 44 ± 2 °C for treating GA, we conducted a pilot randomized controlled evaluator-blinded trial (AF-S0P-07-1.1-01). Eligible participants were patients with histopathologically confirmed GA who had not received any effective local or systemic therapies for at least 3 months. Key exclusion criteria comprised severe infection, immunodeficiency, or malignancy. A total of 8 enrolled patients were randomly assigned to either the treatment group, which received localized hyperthermia using an infrared device (Liaoning Yanyang Medical Equipment Co, Ltd), or the control group, which underwent a sham procedure involving all steps except for the actual heating. The most recently emerged lesion was designated as the target for treatment and assessment. The therapeutic regimen consisted of weekly sessions over the first 3 months, continuing until complete GA resolution or until the 3-month endpoint was reached. All patients were followed up until the sixth month after the initial treatment.

This study enrolled 8 patients (4 per group), all of whom completed the 6-month follow-up. No significant differences existed in lesion count, target lesion area, or prior treatments (Table I).

**Table I:** Baseline characteristics and treatment outcomes of participants

Characteristic/outcome	Intervention (*n* = 4)	Control (*n* = 4)
Baseline characteristics		
Age, mean ± SD (y)	49.8 ± 14.6	36.3 ± 19.0
Female, *n* (%)	2 (50%)	2 (50%)
GA subtype, *n* (%)		
Localized (LGA)	2 (50%)	1 (25%)
Generalized (GGA)	2 (50%)	2 (50%)
Patch-type (PGA)	0	1 (25%)
Disease duration, mean ± SD (mo)	26.3 ± 7.5	36.3 ± 18.8
Target lesion area, mean ± SD (cm^2^)	9.4 ± 13.1	17.5 ± 26.9
Lesion count, mean ± SD	9.5 ± 9.7	5.0 ± 4.3
Treatment outcomes		
Cure rate at 3 mo, *n* (%)	3 (75%)	0 (0%)
Median time to cure (wk)	4 (range: 3-9)	16 (1 case)
Lesion count vs baseline at 3 mo	0/1, 0/5, 20/26, 0/6	1/1, 5/5, 12/12, 2/2
Lesion count at 6 mo	0/1, 0/5, 13/26	0/1, 5/5, 12/12, 2/2

Due to the small sample size (*n*= 8), no formal statistical hypothesis testing was performed. Data are presented descriptively.

*GA*, Granuloma annulare; *GGA*, generalized granuloma annulare; *LGA,* localized granuloma annulare; *PGA,* patch-type granuloma annulare.

The intervention group demonstrated marked clinical improvement, with 3 patients achieving complete response (Case 1: penile lesion resolved in 4 weeks; Case 2: pedal lesions resolved by 9 weeks despite prior steroid failure; Case 4: hand/trunk plaques cleared by 3 weeks with concurrent regression of satellite lesions) and 1 generalized GA patient showing partial response (50% reduction in lesion count) (Fig 1). In contrast, the control group exhibited minimal therapeutic response, with only 1 patient achieving delayed clearance at 16 weeks and 3 showing no response. Photographic documentation confirmed progressive centrifugal fading of annular borders and resolution of induration without scarring. Quantitatively, the intervention group showed significantly superior outcomes at 3-month follow-up, with higher complete response (75% vs 0%), shorter median time to cure (4 weeks vs 16 weeks), and a 2.4-fold reduction in lesion count from baseline compared to unchanged controls, with no recurrences observed during follow-up (Table I). The treatment demonstrated a favorable safety profile, with a single device-related blister resolving spontaneously and 100% protocol completion, though the small cohort precludes definitive assessment of rare adverse events.

**Fig 1:**
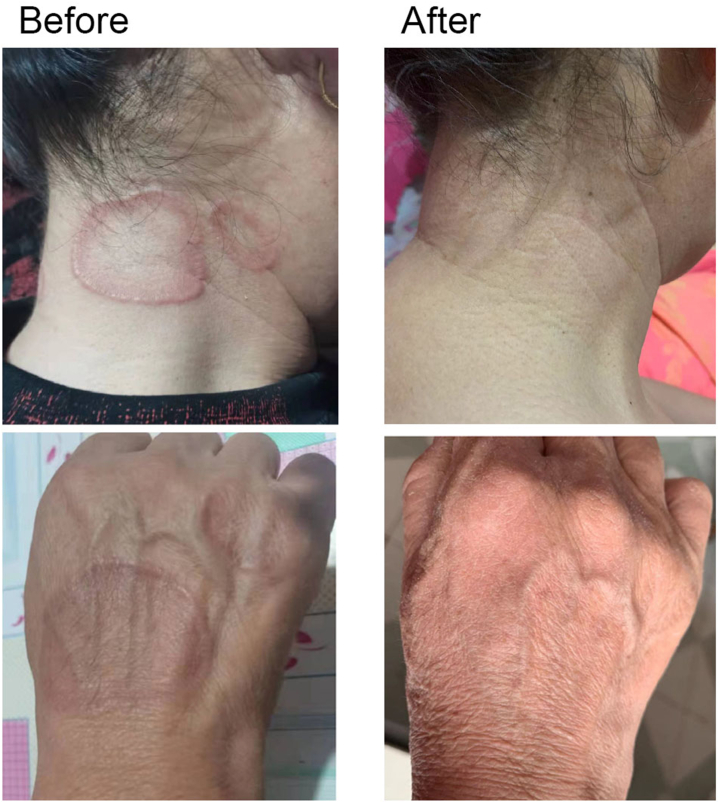
Treatment course of 1 generalized GA patient before and after 12 treatment sessions. *GA*, Granuloma annulare.

This first randomized controlled study demonstrates localized hyperthermia's potential for GA, showing a 75% cure rate at 3 months without conventional side-effects. The mechanism may involve heat-mediated immunomodulation, and despite a small sample size, the promising safety and efficacy support further optimization and larger trials.

### Declaration of generative AI and AI-assisted technologies in the writing process

We used AI tool DeepSeek to polish the language.

## Conflicts of interest

None disclosed.
